# Notes on the United States of North America, during a Phrenological Visit in 1838-9-40

**Published:** 1843-01

**Authors:** 


### ART. IV.

*Notes on the United States of North America, during a Phrenological Visit in* 1838-9-40. By GEORGE COMBE. Three Volumes.—*Edinb.* 1842. 8vo, pp. 371, 400, 488.

MR. COMBE in the introduction to this work, when discussing the difficulties which beset any single observer who endeavours to describe a great nation, says, with truth and acuteness, that each writer should be regarded as a single witness in a vast and complicated cause, and to arrive at truth, the reader must analyse and compare the various reports. Each traveller has probably moved in a different sphere, and from original difference of mind, of education, and of pursuits, has observed and been interested in different objects. This view is particularly applicable to Mr. Combe's own "Notes;" and we gladly accept him as a witness: for although he is not a medical man, yet his favorite pursuit has led him to observe and to study the reciprocal influence of the body and the mind, and the great causes which produce their healthy and diseased conditions. Mr. Combe visited the United States, and resided sometime in New England, for the purpose of spreading the knowledge of phrenology by lectures. He was consequently thrown much into the company of those who, with intellectual tastes, active, practical minds, and with little leisure from the business of life (a numerous class in America), are willing to increase their knowledge by listening to viva-voce communications. Mr. Combe appears not merely to have imparted his own knowledge, but to have observed closely those who came to him for information, and in his "Notes" he has recorded his impressions of the mental constitution of remarkable individuals, and of the different classes of the community, clothed in phrenological language, as well as many observations on manners, habits, ventilation, public institutions, prisons, lunatic asylums, and other such topics connected with health and disease. We shall analyse or extract such of these passages as most interest us, as medical observers, passing wholly by the "Notes" on religion, education, and politics, which form the great bulk of these volumes.

1. *Notes on habits, climate, &c.* The dryness of the air of the United States, when compared with our moister island climate, has often been remarked, and the fact that the American women lose their bloom much sooner, and the men become in appearance withered and old more speedily than in this country, has been partly explained on this supposition. Mr. Combe found the climate of New England much more stimulating (so that wine was unnecessary), the air drier, and apparently more highly charged with electricity. "The habitual state of the American people, also, is one of much higher mental excitement than that of the inhabitants of Britain." (Vol. i. p. 79.)

Before the commencement of winter the weather generally is calm, mild, and dry, the sun shining without clouds the whole day, and a gentle haze pervading the whole sky from the stillness of the air. This is called the Indian summer, and is said to be the most delicious season of the year in the United States. (p. 159.)

Mr. Combe was especially struck with the deficient ventilation in public buildings and rooms in which meetings were held. He often strongly called the attention of his audiences to the dependence of the mind on the state of the brain; to the dependence of the brain on the blood which circulated through it; and to the dependence of the blood on the respiration and digestion, thus showing the "direct connexion between pure and sound digestion and mental vigour." The rapid transition from ill-ventilated rooms and churches, of a temperature from 70° to 80° (F.) to the cold air, 5° or 10° below zero, must, Mr. Combe thinks, be a fruitful cause of phthisis so prevalent in the United States. "I sat," says Mr. Combe, "three hours in the gallery of the senate chamber to-day, and afterwards experienced those debilitating, irritable, and unpleasant sensations which are generated by imperfectly decarbonized blood." (p. 95.)

Mr. Combe is in accordance with other travellers as to the beauty of the younger American ladies, but "their forms are fragile, and indicate liability to premature decay." (p. 112.) He quotes Dr. Lindsay, of Washington, who says that all Europeans who have visited the United States agree that the women are inferior in vigour, strength, and robustness to corresponding classes in Europe, and especially in Great Britain. "The European woman has a much more florid and healthful complexion, a much more vigorous person, and is capable of enduring much more fatigue and exposure, and of performing much harder labour. The slender, and delicate, and fragile form, the pale, sallow, and waxen complexion, which are so common among us, are comparatively seldom seen abroad." Dr. Lindsay instances the long walks which English ladies are in the habit of taking as a proof of their superior vigour. A walk of ten miles would be an event to be talked of for life by an American lady! Dr. Lindsay thinks that his countrywomen are not only less robust but more liable to ill health, particularly from derangement of the digestive and nervous system.

"They rarely," says Mr. Combe, "walk abroad for fresh air or exercise. In general they live and sleep in ill-aired apartments. Their duties press constantly on their minds, and they do not give sufficient effect to the maxim, that cheerful amusement and variety of occupation are greatly conducive to health. They do not properly regulate their diet; pies, pastry, and animal food are consumed in quantities too abundant for a sedentary life; and baths and ablutions are too rarely used." (Vol. ii. p. 127.)

Mr. Combe seems to have endeavoured to attract the attention of ladies to the value of health, by showing them in his lectures the connexion between beauty, in the proportions and forms of the whole of the body, and health, recommending to the fair part of his auditory the study of the human figure in statuary and in painting, as the ideal models of perfect development; so that if the mother saw "the abdomen of her child tending to become tumescent, the chest flat, and the head enlarged, she would easily become aware that there was some deviation from the laws of health." This excellent advice was well received in the room, but he was attacked in a newspaper subsequently for offending "the national sense of propriety." This reminds one of Swift's notion, that "a nice man is a man of nasty ideas."

The injurious effects of swallowing the food too hastily is admitted to be exemplified on a large scale among the Americans, owing to their active business-like habits and industry. In New York, at one of the large hotels, 150 persons crammed down their breakfast in fifteen minutes. "You Europeans," said an American to Mr. Combe, "eat as if you actually enjoyed your food." "Assuredly we do," was the reply, "and you Americans will never escape from dyspepsia and headaches until you also seem to enjoy your meals." An excellent hint to all who suffer from the same symptoms in this country. In private life in America the same haste is practised; for all are engaged in active occupations: there is no large idle class as in the older countries, and the few who are independent of work seem to be in a false position.

"If a young man inherit a fortune and follow no profession, it generally happens that in less than ten years he ruins his fortune in low pursuits. In a few years more his health is equally reduced with his estate, and he is banished from society. These young men are pitied, their fate is predicted, and the prophecies are too generally realized. The few who form exceptions are men who engage in literature or science as a pursuit." (Vol. ii. p. 231.)

II. *Notes on lunatic asylums, schools for the blind, prisons, &c.* The lunatic asylum at Worcester met with Mr. Combe's approval. Its superintendent physician, Dr. Woodward, was, phrenologically and physically, the ideal of his class. "Large limbs, a large abdomen, large lungs, and a large head." A mixture of all the temperaments, and a head with well-developed animal propensities, but one in which the moral sentiments and intellect predominate, complete the portrait.

"This combination," says Mr. Combe, "produces a powerful and commanding person, characterized at once by vivacity, energy, and softness; and a mind in which intellectual power is chastened by the most kind and cheerful moral dispositions. If that well-spring of spontaneous vivacity which accompanies large lungs and a large brain be wanting, the individual will be more apt to sink under the depressing influences which the diseased minds of his patients will exert over his own, than to excite their faculties to more healthy and agreeable action. If he be deficient in the moral organs of the brain, he will want sympathy, softness of expression, and justness of feeling; while if he be deficient in the reflecting intellectual organs, he will want sagacity to trace effects to their causes and to discriminate character; or if the deficiency be in the observing organs he will lack the power of attention to incidents and details." (Vol. i. p. 57.)

In this asylum ventilation is carefully attended to. The warm air is conveyed into the galleries by pipes, which open near the ceiling, and from thence into the cells which communicate with the gallery, by openings above the doors; as it is found that warm air diffuses itself more agreeably than when the pipe opens at the floor. Each cell has an aperture into an air-chimney, and all the air-chimneys open into a vast garret with numerous windows for letting out the noxious air: by this plan the air-chimneys are protected from the wind, which otherwise is apt to descend in cold weather. The patients are treated on the humane system. A ball is given at Christmas, to which they look forward with great pleasure. One fourth are allowed to go into the village on errands unattended, and only one man has escaped. "Social parties are given from time to time, with music and dancing, which, with religious worship on Sundays, have an excellent effect on the minds of the patients." The music is performed by them. Dr. Woodward thinks that more than an average number of shoemakers are insane, owing to their ill-ventilated rooms and shops, and unfavorable position whilst working. He thinks that there should be asylums for drunkards; regarding intemperance as a physical disease, generally curable, but that confinement is almost essential to prevent the temptation until the patient is cured.

No one will deny that confinement would be the greatest blessing to a confirmed drunkard; but how is the line to be drawn between those who have lost entirely their command over their will, and those who by strong mental exertion might reclaim themselves? The exertions of Father Matthew and, in a minor degree, of temperance societies, show that, at least, if drunkenness is a physical disease, it is one of those diseases which the mind can control: and in the present state of society we cannot make people virtuous on compulsion, however desirable it might be.

The condition of lunatics in America, as in this country, is still capable of great amelioration. In a return of the lunatics in the state of New Hampshire in 1838, some were "in cages and cells, some in irons and chains, and some in jails." (p. 197.) At a new pauper lunatic asylum near New York, and under the management of the corporation of that city, Mr. Combe was surprised to find not the slightest arrangement had been made for ventilation; and there was a similar deficiency in an orphan-school under the same management.

"The consequences are visible in the appearance of the children: many of them are suffering under ophthalmia, and they present generally that sunken, inanimate, and unhappy aspect which betokens blood in a bad condition from imperfect nutrition and impure air. There is, I believe, no stinting of food; but the digestive functions suffer from the confinement in an unwholesome atmosphere, and hence the nutrition is imperfect." (Vol. i. p. 226.)

One cause of this is in the political condition of the country. "The poor, the insane, and the criminal have few, and these not noisy, advocates, and their interests are postponed;" and besides, "it is an unpopular duty to expose the imperfections of any American institution." (Vol. i. p. 227.)

Mr. Combe was informed that the average of insanity is higher among Quakers than among the general community, for two reasons: "First, their doctrine of the workings of the Holy Spirit, and their inward light, their narrow circle of interests, and limited education act unfavorably on minds predisposed to disease. Secondly, they intermarry extensively within close degrees of consanguinity." (Vol. ii. p. 144.) The second is in accordance with the well-known fact, proved by breeds of cattle, (where there are no such doubts as to the genuineness of the breed as may often vitiate our conclusions regarding similar effects in our own species,) that breeding in-and-in impairs the physical perfection in the offspring; but that a narrow circle of interests and a limited education cause insanity in those predisposed to disease, is a statement contradicting well-ascertained facts. The less highly a people or a class are educated, and the more primitive, simple, and tranquil the state of society, the less numerous are the insane. Insanity increases in the ratio of (what is called) high civilization. And instead of agreeing with Mr. Combe as to the effects on the health of the peculiar doctrines of this sect, our observation has rather led us to think that the doctrine of an inward light has induced in such Quakers as sincerely and heartily embrace it, a serene tranquillity of mind, such as philosophers have aimed at; a quiet mental condition, the opposite to that extreme sensitiveness, irritability, mobility, and morbid restlessness of mind which is too often the hereditary curse of those who are tainted with insanity, and consequently a condition which, in the way most suitable to the individual's own mind, should be assiduously cultivated and encouraged by all those who are unhappily predisposed to this disease. Besides, Mr. Tuke has shown the fallacy of the same opinion, formerly prevalent in England.

The effect of solitary confinement upon the brain, according to the physicians of the New Jersey State Prison, is to enfeeble its whole functions: whilst such solitude paralyses the evil propensities, from want of exercise, it equally debilitates the rest of the mind. The prisoners often lose their acuteness and become simpletons. Even when well administered Mr. Combe thinks it injurious, as it is not in harmony with a sound knowledge of the physiology of the brain:

"According to my view of the laws of physiology this discipline reduces the tone of the whole nervous system to the level which is in harmony with solitude. The passions are weakened and subdued, but so are all the moral and intellectual powers. The susceptibility of the nervous system is increased, because organs become susceptible of impressions in proportion to their feebleness. Hence it may be quite true, that religious admonitions will be more deeply felt by prisoners living in solitude than by those enjoying society; just as such instruction, when addressed to a patient recovering from a severe and debilitating illness, makes a more vivid impression than when delivered to the same individual in health; but the appearances of reformation grounded on such impressions are deceitful. When the sentence is expired, the convict will return to society with all his mental powers, animal, moral, and intellectual, increased in susceptibility, but lowered in strength." (Vol. ii. p. 13.)

This weakening of the faculties is observed in all those who have been in the prison more than a year; if the confinement is continued longer, "the most accomplished rogue," says the Report, "will lose his capacity for depredating with success upon the community; if still a rogue, he is one who may be easily detected."

III. *Notes on Phrenology.* Mr. Combe's fixed idea is phrenology, and through its medium he looks at and speculates upon men, manners, politics, religion, and every subject connected with humanity. Much that is common-place, many musings, very evident and unmistakable observations which would not pass in their every-day garments, give a fresh impression of novelty from the unusual clothing in which they are dressed; and this freshness of impression is by no means an unpleasing one. But this is not all. Mr. Combe is an observing and reflecting man, and the results are everywhere visible. Phrenology (independently of its organology or craniology) must be admitted to be a readily-comprehended and easily-applicable classification of the mental powers; a system which, from its practical nature, is calculated to interest a greater number of minds than more abstract metaphysical speculations. It is a generalization of a large number of facts relating to the faculties of the human mind, such as must be of great interest to every inquirer after truth. The intimate connexion between mind and the body is duly recognized. Physiology and psychology are not divided; but the influence of the temperament of the individual, his state of health, and the action of the air, climate, diet, &c. upon his mental manifestations, are insisted upon. Mr. Combe observes that he met with many acute men in America who understood the metaphysics of phrenology, but were ignorant of the situation of a single organ; and from our own observation we should imagine this also much the case in this country also: many who think themselves incompetent to decide the physiological truth of the system, yet willingly make use of it as a convenient practical adaptation of metaphysics to the elucidation of character.

The following sketch of the temperaments, which Mr. Combe presented in his lectures, gives the leading characteristics of four marked peculiarities of our species in so short a compass, that we cannot forbear quoting it:

"Where the brain is large, and the lungs (as indicated by the chest,) and the abdomen small, the nervous temperament is present, accompanied by fine hair, a fine texture of the skin, great mental activity, and an aversion to muscular motion. When the lungs are disproportionately large in relation to the brain and abdominal viscera, the sanguine temperament is indicated by a fair or reddish hair, blue eyes, and a ruddy countenance, by a great love of muscular action, and also by mental activity, manifested in *action* rather than in study, thought, and composition. When the abdominal region predominates over the brain and lungs the lymphatic temperament, characterized by an unwieldly figure, coarse fair hair, a sleepy eye, and a heavy inexpressive countenance, is present, and then the cerebral action is low, and the mental faculties inactive. The bilious temperament is indicated by a dark skin, dark strong hair, a harshly-expressed outline of the countenance, and a firm compact condition of the muscular system, with great powers of sustained action—either mental or corporeal—corresponding to the quality expressed by the word 'bottom,' when applied to horses." (Vol. i. pp. 127-8.)

"Of ten gentlemen present at this lecture, six presented the combination of the nervous and bilious, two or three the nervous, sanguine, and bilious, and scarcely in one was any trace of the lymphatic to be found."

This certainly corresponds with the mental and bodily activity of the New Englanders, from whom Cæsar would with difficulty have selected his court when he wished for "Men about him that are fat, sleek-headed men, and such as sleep o' nights."

Boston is perhaps the most intellectual city of New England, which was peopled, as it will be recollected, by those who left their own country to enjoy more complete religious liberty. The female heads of Boston are remarkable for their beautiful development of the moral and intellectual departments. "In the men, also, large moral and intellectual organs are very general; but benevolence and veneration are more frequently large than conscientiousness." (p. 131.) The persons who composed Mr. Combe's class were the élite of the city.

Mr. Combe's ruling passion sometimes makes his book very diverting. He goes to a sailors' church to hear Mr. Taylor, and after giving us the text, at full length, this is the next comment: "His temperament is bilious-nervous; the anterior lobe of his brain is long and high, but not broad;" and then a list of his organs. Many children were brought to Mr. Combe for examination with less cautiousness and more self-esteem than in Scotch children, with very large acquisitiveness. Where the heads were large and the chests small he most judiciously recommended their parents to limit the mental exertions and increase the bodily activity of the children; advice which cannot be too often repeated and urged upon parents, who by neglecting it either shorten the duration of their children's lives, losing them just when they are most promising, or if unfortunately they survive to maturity these early promises are broken, because the feeble body is incompetent to that persevering and continuous action which successful mental exertion imperatively requires. Mr. Combe mentions (p. 168,) having seen the common-place book of a young man at Worcester, who had an extraordinary talent for languages. He was a smith, and worked six, eight, and twelve hours a day at his forge, but in his leisure time he learned to read upwards of *fifty* languages with greater or less ease. "One thing is obvious, that the necessity of forging saved this student's life. If he had not been forced by necessity to labour he would, in all probability, have devoted himself so incessantly to his books, that he would have ruined his health, and been carried to a premature grave." (p. 200.)

The following observations on the character of the Indians and the Negroes are of interest to the man of science as well as to the politician. The North American Indians are extirpated by war, but never reduced to servitude:

"The development of their brains shows large organs of destructiveness, secretiveness, cautiousness, self-esteem, and firmness, and greater benevolence, conscientiousness, and reflection. This indicates a natural character that is proud, cautious, cunning, cruel, obstinate, vindictive, and little capable of reflection or combination. The brain of the Negro shows proportionately less destructiveness, cautiousness, self-esteem, and firmness, and greater benevolence, conscientiousness, and reflection than the brain of the native American. The negro is therefore naturally more submissive, docile, intelligent, patient, trustworthy, and susceptible of kindly emotions, and less cruel, cunning, and vindictive than the other race." [Hence the difference in their fates—the untameable free-man and the tame slave.] "In both the brain is inferior in size—particularly in the moral and intellectual region—to that of the Anglo-Saxon race; and hence the foundation of the natural superiority of the latter over both: but my conviction is, that the very qualities which render the Negro in slavery a safe companion to the white, will make him harmless when free; the fears, therefore, generally entertained of his commencing, if emancipated, a war of extermination or for supremacy over the whites, appear unfounded: unless, after his emancipation, the whites should commence a war of extermination against him." (Vol. ii. p. 79.)

Much of Mr. Combe's work consists of phrenological speculations on politics, education, and religion. Those readers who are not phrenologists may be prejudiced against the system, from the author's constantly bending phrenology to his own political, moral, and religious theories; but the principle should always be borne in mind, that theories are not the facts themselves, but merely attempts to explain, or generalize, or deduce from the actual facts, and that the theory may be wrong whilst the facts on which it was founded remain true. But however his readers may differ with him in their creeds, they must admit his candour and his fairness. He states generally both sides of a question, putting strongly and forcibly the arguments of those whom he thinks wrong.

His views and observations may in some instances be incorrect, one-sided, and prejudiced; but he gives the impression that he believes them to be true, that he is firmly bent on promoting the general happiness of mankind, and as firmly convinced that his plan is the right one.

When with this fairness and candour are combined active powers of observation and of reflection, aided by earnestness and indefatigable industry in pursuing an object, the writings of such a man must contain matter for reflection; and we cordially recommend him as a witness whose evidence is worth weighing by all those interested in the habits, customs, politics, and religion, as well as in the "phrenological developments" and physical condition of our transatlantic brethren.

One omission in these volumes is highly creditable to their author, especially as the study of individual character is his favorite pursuit. There is an entire absence of any personal reflections which, although they might amuse many readers and exhibit the writer's own cleverness, and enhance the popularity of his work, might also wound the feelings of an individual. And there is the same absence of ill-natured or satirical observations on habits, manners, or institutions with which so many clever accounts of America are so disgracefully disfigured. From the exhibition of these petty feelings—the indulgence in which is as injurious to the persons who cultivate them as to those who are sensitive enough to be annoyed by them, and which if such people cannot help indulging they should, at least, restrict to their own circle or small coterie, instead of endeavouring to set nations at variance—from such feelings science is very free. There is no disposition here to look with envy or with ill-nature at the progress of science in other countries; but, on the contrary, a tendency to give to foreigners their full and fair meed of praise. And, indeed, although female satirists or writers of piquant books of travels may produce much effect on the students of circulating libraries and of small provincial book-clubs, by their ephemeral superficialities, yet the common sense of England is not to be judged of by such converts. Such books, whatever our transatlantic neighbours may think, are not the text-books here of those whose opinions are worth possessing—of those who candidly look for truth, and who are not to be deluded into the belief that vanities, trivialities, foibles, and vices, from being dressed up in garments different to those in which they are in the daily habit of seeing them clothed at home, are on this account the peculiar fruits of another soil. They feel that the real questions at issue lie much deeper than the mere surface which these scribblers have looked at and attempted to picture as the whole substance. It is to be hoped that the more intimate union which science, by means of steam, has so recently effected, is but the commencement of a more intimate connexion between two great families so nearly related. The greater frequency of intercourse between the leading men of both countries must soften prejudices, and dissipate misconceptions and errors which distance, ignorance, and false witnesses have produced. And we are sure that science, which "emollit mores, nec sinit esse feros," will not be wanting in strengthening a connexion which, as it is for the happiness and prosperity of both parties, as well as for the whole world, should be close and perpetual.

